# Patient safety in nursing care during medication administration^1^

**DOI:** 10.1590/1518-8345.2350.3017

**Published:** 2018-08-09

**Authors:** Júlian Katrin Albuquerque de Oliveira, Eliana Ofélia Llapa-Rodriguez, Iza Maria Fraga Lobo, Luciana de Santana Lôbo Silva, Simone de Godoy, Gilvan Gomes da Silva

**Affiliations:** 2 MSc, RN, Serviço de Controle de Infecção, Hospital de Urgência de Sergipe, Aracaju, SE, Brazil.; 3 Post-doctoral fellow, University of British Columbia, Columbia, Vancouver, Canada. Adjunct Professor, Departamento de Enfermagem, Universidade Federal de Sergipe, São Cristóvão, SE, Brazil.; Universidade Federal de Sergipe, Departamento de Enfermagem, Universidade Federal de Sergipe, São Cristóvão, SE, Brazil; 4 PhD, Physician, Núcleo de Epidemiologia, Segurança do Paciente e Infecção Hospitalar, Hospital de Urgência de Sergipe, Aracaju, SE, Brazil.; 5 Master’s student, Universidade Federal de Sergipe, São Cristóvão, SE, Brazil. Assistant Professor, Estácio, Aracaju, SE, Brazil.; Estácio, Aracaju, SE, Brazil; 6 PhD, Professor, Escola de Enfermagem de Ribeirão Preto, Universidade de São Paulo, PAHO/WHO Collaborating Centre for Nursing Research Development, Ribeirão Preto, SP, Brazil.; 7 Undergraduate student in Nursing, Universidade Federal de Sergipe, São Cristóvão, SE, Brazil. Scholarship holder at Programa Institucional de Bolsas de Iniciação Científica, Coordenação de Pesquisa, Universidade Federal de Sergipe (PIBIC-COPES), Brazil.

**Keywords:** Central Venous Catheter, Patient Safety, Nursing Care, Infusions, Intravenous, Infection Control, Quality Management

## Abstract

**Objective:**

to evaluate the conformity of care practices of the nursing team during the administration of drugs through central vascular catheter.

**Method:**

a descriptive, prospective, observational study conducted in an Intensive Care Unit. The non-probabilistic intentional sample consisted of 3402 observations of drug administrations in patients with central vascular catheters. The previously validated collection instrument was constructed based on the Guideline for Prevention of Intravascular catheter-related infections. Data was collected through direct observations of nursing practices performed by the nursing team. The analysis used analytical, descriptive and inferential statistics (Chi-square test and Fisher’s exact test).

**Results:**

a total of 3402 procedures of drug administrations were observed. Female nursing technicians performed the highest number of actions. In none of the procedures did the professional perform all necessary actions. 0.2% of drug administrations were preceded by hand hygiene and 1.3% by disinfection of the multidose vial, ampoule or injectors.

**Conclusion:**

the practice evaluated was classified as undesirable. Failure to achieve the desired conformity was probably due to the low adherence of professionals to the practice of hand hygiene and disinfection of materials, injectors and connectors.

## Introduction

The technologies used in the daily work in health institutions are fundamental for the development of care. However, they challenge professionals and managers to maintain the quality of care processes, especially in intensive care units, where a greater number of technologies is added, named: soft, hard-soft or hard^1^.

As an attempt to achieve quality in the care provided, the management of these technologies and care processes becomes a priority in health care units^2^. In this context, the Intensive Care Unit (ICU) is considered a technological environment, with assistance offered in different degrees of complexity and with factors that may lead to the occurrence of adverse events (AE) that can compromise patient safety^3^.

In the United States of America, approximately 25% of hospitalized patients have a central vascular access placed, which is even more common among patients admitted to the ICU. In general, central venous catheters (CVCs) are used to supply energy, water and electrolyte needs, to collect blood, to administer medication and for hemodynamic monitoring. However, the use of CVCs may also be associated with the occurrence of complications, among which are bloodstream infections^4^ ^-^ ^6^.

In general, to ensure patient safety, good practices implemented by practitioners must be imperative. However, there is low adherence of the multi-professional team to preventive measures for AEs, which compromises the quality of care and makes it insecure, especially regarding medication administration^7^ ^-^ ^8^.

The nursing team has a fundamental role in the reduction of these AEs, since it works uninterruptedly in care and represents, in most cases, the highest percentage of workers in health services. In addition, the nursing team is more involved in the management of vascular accesses during drug administration and dressings^5^.

A safe health care is the obtaining of greater benefits with low risks to the population. In this sense, the care must be in line with the resources available and the existing social values and it should be undertaken through the analysis of the structure, the work processes and the structure of care. The evaluation of the assistance includes the unceasing search for identification of shortcomings in the procedures and practices which organize the actions, leading to improvement in the processes and results, considering the conformities established by the regulatory bodies and the satisfaction of the service users^9^.

The protocols and recommendations regarding best evidence are not able to solely modify behaviors and influence good practices for the control of infections. Therefore, interventions and evaluations of care practices are necessary to verify if preventive actions are being carried out effectively^10^.

To evaluate the conformity of the care process, Carter’s Positivity Index (PI) can be used to classify care into quality categories, such as desirable, adequate, safe, borderline, undesirable or poor^11^ ^-^ ^12^.

Given the above and the importance of quality care associated to safe practices, especially in the administration of medications through the CVC, and taking into account the gap in the scientific literature on the subject, the following question was considered pertinent: how is the care process carried out by the nursing team during drug administration through the CVC in an intensive care unit? In this sense, the research aimed to evaluate the conformity of the nursing team’s care practice during the administration of drugs through a central vascular catheter.

## Method

A descriptive, prospective and observational study was carried out in an adult intensive care unit of the largest public hospital and the main gateway to the Unified Health System (SUS) for cases of high complexity in the state of Sergipe, Brazil. The unit has 27 beds and a team with 23 nurses and 80 nursing technicians.

In order to calculate the sample size, a previous survey was carried out during seven days to determine the daily mean of the procedure observed^13^ ^-^ ^14^. A significance level of 95% was considered, with a margin of error of 5%.

The non-probabilistic intentional sample consisted of 378 drug administrations performed by nursing professionals. The inclusion criteria were intravenous administration of drugs in patients with central vascular catheters, performed by nursing professionals who had been working at the ICU for at least six months.

An instrument was elaborated based on the recommendations of the *Guideline for Prevention of Intravascular catheter-related infections* ^15^. The form was divided in two parts. The first one addressed the characteristics of the vascular catheter (location, composition and duration of device use) and of the nursing professionals (professional category, gender and work shift). The second part contained the nine actions to be observed during the administration of drugs and items to record the availability of equipment and supplies needed to carry out each practice. 

The registration of the actions was done according to four response options (yes, no, does not apply (NA) and no record (NR)). The assistance was considered in conformity when the recorded situation was “yes” or NA and not in conformity when the answer was “no” or NR. 

A pilot test was conducted to validate the instrument, verifying if it answered to the research objectives^13^ ^-^ ^14^. The results of the pilot test were not part of the final findings of the present study.

Two observers participated in the data collection. For the development of this activity, they underwent previous training and were evaluated through a specific test. The Kappa test showed an agreement of 0.927, an almost perfect agreement for all the procedures observed. 

Data was collected from January to March 2016 through direct observations of professionals during the administration of medications. The schedules for the observations were defined considering the periods in which the greatest number of procedures was carried out, a context identified during the pilot test and confirmed by the management of the service. Thus, the observations were carried out in three distinct periods: from 8am to 11am, from 2pm to 5pm and from 7pm to 11pm. 

It should be noted that in each work shift, before starting the observations, the researchers verified the availability of all the necessary material for the execution of the procedures, among them: glove, mask, parenteral infusion equipment, hand sanitizer (soap/alcohol-gel), alcohol (70% alcohol) for the disinfection of ampoules, tapers and injectors of parenteral solutions.

For the analysis of the process indicators, the general and specific conformity rates were calculated for further determination of the quality of care according to Carter’s PI^16^. Thus, 100% positivity indicates desirable care, 90-99% adequate care, 80-89% safe care, 70-79% borderline care and less than 70% undesirable or poor care. 

The association between variables (professional category, work shift and gender) and the procedure was also carried out. As inferential tools, the Chi-square test and the Fisher’s exact test were used, adopting a significance level of 5%, in which the relation is significant when p-value <0.05. Statistical analyzes were performed using the free R software, version 3.2.3. 

Nursing professionals who accepted to participate in the study signed the Informed Consent Form (TCLE). It should be noted that for the reduction of bias in the observations, such as behavior alteration and acting, the form with the specific actions evaluated was not presented to the participants.

The project was approved by the Research Ethics Committee of the Federal University of Sergipe, with the number of the Certificate of Ethical Assessment 50544115.9.0000.5546, respecting Resolution 466/2012, on July 20, 2015. It should be noted that patients hospitalized in the Unit investigated did not suffer interventions during the study and there was no interruption or negative implications in the assistance offered. Therefore, the research did not pose any risks to these patients.

## Results

A total of 3402 actions were observed during the administration of medications in patients using a central vascular catheter. The observations corresponded to 378 procedures performed by nursing professionals (Table 1).

**Table 1:** Characterization of nursing professionals observed during medication administration. Aracaju, SE, Brazil, 2016

Variable	Shift n= 378			
	Morning n(%)	Afternoon n(%)	Night n(%)	
Procedures performed by nursing professionals according to gender				
Male	28 (7.4)	30(7.9)	15(4.0)	Mean 24.3 Median 28 SD* 8.14
Female	98(25.9)	96(25.4)	111(29.4)	Mean 4.7 Median 98 SD* 8.14
Procedures performed according to professional category				
Nurse	5(1.3)	3(0.8)	6(1.6)	Mean 4.7 Median 5 SD* 1.53
Nursing technician	121(32)	123(32.5)	120(31.7)	Mean 121.3 Median 121 SD* 1.53

Source: Research data

*SD= Standard Deviation

Most of the observations were of double lumen catheters, with up to 7 days of duration and located in the right subclavian vein.

We highlight A3 (98.6%) and A8 (97%), classified as adequate, and A6 (87.5%), classified as safe, presented the best conformity rates, as shown in Table 2.

**Table 2:** Distribution of the nine actions observed according to occurrence and specific conformity rate. Aracaju, SE, Brazil, 2016

Actions observed	Sample n=3402	Actions in conformity N	Specific rate %
A1. Performs hand hygiene.	378	5	1.3
A2. Disinfects the multidose vial, ampoule or injector of the serum vial with alcohol solution.	378	6	1.5
A3. Uses sterile syringe and needle to prepare the medication.	378	373	98.6
A4. Disinfects the injector of the serum before the introduction of the equipment.	378	209	55.2
A5. Performs hand hygiene after preparing the medication.	378	1	0.2
A6. Uses procedure gloves.	378	331	87.5
A7. Disinfects the injector or taper with alcohol solution before introducing the medication.	378	161	42.5
A8. After administration, discards the syringe and needle in the sharps bin.	378	367	97.0
A9. Performs hands hygiene after the procedure.	378	122	32.2

Source: Research data

Actions involving hand hygiene (A1, A5 and A9) during drug administration and disinfection of ampoules, vials and injectors with 70% alcohol (A4 and A7) were characterized as undesirable or poor, with specific conformity rates of less than 70%.

In table 3, the action A5 (p=0.0370) stands out because it is significant. In addition, the category Nursing Technician presented a higher number of actions in conformity when compared to Nurses.

**Table 3:** Association between the professional category variable and the medication administration procedure. Aracaju, SE, Brazil, 2016

Actions observed	Classification	Actions according to professional category n=3402		p-value significant (<0.05)
		Nur n (%)	Tech n(%)	
A1. Performs hand hygiene.	C*	-	5 (1.3)	1.0000
	Nc^†^	14 (3.7)	359 (95)	
A2. Disinfects the multidose vial, ampoule or injector of the serum vial with alcohol solution.	C*	-	6 (1.6)	1.0000
	Nc^†^	14 (3.7)	358 (94.7)	
A3. Uses sterile syringe and needle to prepare the medication.	C*	13 (3.4)	360 (95.2)	0.1728
	Nc^†^	1 (0.3)	4 (1.1)	
A4. Disinfects the injector of the serum before the introduction of the equipment.	C*	7 (1.9)	202 (53.4)	0.8951
	Nc^†^	7 (1.9)	162 (42.9)	
A5. Performs hand hygiene after preparing the medication.	C*	1 (0.3)	-	0.0370^‡^
	Nc^†^	13 (3.4)	364 (96.3)	
A6. Uses procedure gloves.	C*	11 (2.9)	320 (84.7)	0.3968
	Nc^†^	3 (0.8)	44 (11.6)	
A7. Disinfects the injector or tamper with alcohol solution before introducing the medication.	C*	5 (1.3)	156 (41.3)	0.7987
	Nc^†^	9 (2.4)	208 (55)	
A8. After administration, discards the syringe and needle in the sharps bin.	C*	13 (3.4)	354 (93.7)	0.3435
	Nc^†^	1 (0.3)	10 (2.6)	
A9. Performs hands hygiene after the procedure.	C*	5 (1.3)	117 (31)	1.0000
	Nc^†^	9 (2.4)	247 (65.3)	

Source: research data

*C - action in conformity; †Nc- action not in conformity

The association between the variable work shift and the medication administration procedure showed action A4 (p=0.0210) as significant, with the highest conformity rate during the night shift (40.8%). On the other hand, the action A7 (p=0.0166) showed better conformity during the morning (34.5%) and afternoon (37.7%). 

The actions A2 (p=0.0142) and A8 (p=0.0013) were significant in the association between gender and the procedure observed. The highest conformity rate in action A2 was of males, with 66.6%. In action A8, the female nursing professionals showed greater adherence to the practice, of 88%.

The results showed shortcomings in the medication administration procedure. In addition, there was no professional that complied with all the standardized steps for the medication administration procedure. Thus, the overall conformity rate for the procedure was 0%, rated by Carter’s PI as a poor practice.

## Discussion

Nursing professionals are the category with greater involvement in the manipulation of vascular accesses and, consequently, have a greater chance of acting in the prevention of complications^10^. The present study verified that most of the procedures were performed by nursing technicians, and females were predominant in this category.

Corroborating the results of this study, a research developed in an intensive care unit mentioned that nursing assistants/technicians were the most observed in all work shifts, specifically during the replacement of the infusion system (67.0%), blood collection (69.0%) and medication administration (68.0%)^17^.

According to process indicators, the results of this research evidenced a care classified as undesirable, that is, the professionals did not perform all necessary actions in any of the practices. Conversely, other studies showed conformity rates greater than 80% in the care practices developed by the nursing team^18^ ^-^ ^19^. In the present study, the actions that had low adherence involved the disinfection of materials, injectors and invasive devices and hand hygiene practices.

In this same direction, a study identified a conformity rate below 49% regarding the disinfection of hubs and connectors in all work shifts^17^. Additionally, another study evidenced that in most of the procedures the recommendations regarding the previous disinfection of lateral injectors with 70% alcohol were not followed^20^. However, an Australian study designed to monitor conformity with disinfection of injectors found a 60% conformity^21^. 

Given these results, it is evident that the disinfection of devices with 70% alcohol is still not part of the routine of nursing professionals, as other preventive actions evaluated in this study. According to observations in the context researched, probable causes for this fact can be: forgetfulness, lack of standardization of institutional norms, weaknesses in knowledge, accessibility to protocols and manuals and lack of scientific information about the impact of these actions on hospital infection rates, which generate disbelief in their relevance among professionals.

The disinfection of injectors and devices with alcohol solution is an important action for the prevention of contamination of central catheters and of consequent bloodstream infections, since there is a risk that the contaminants present on the surface of these devices may be introduced intraluminal during the administration of medications^22^. For this reason, the professionals should be alert and aware of the activities performed during their shift, in order to guarantee the sterility of the equipment and an appropriate disinfection for the safety of the patient.

Direct contact between the infected hands of the professional and the patient is one of the main routes of transmission of microorganisms. Therefore, adherence to hand hygiene practice favors the reduction of adverse events^17^. Studies^17^ ^,^ ^23^ ^-^ ^24^ have shown that, although widely disseminated, hand hygiene practices are not yet within the expected standards. Nursing professionals do recognize the importance of this action, but do not incorporate it into their care practices. Thus, improving adherence to this procedure is a major challenge for the control of healthcare-related infections in various institutions.

The nursing team reports that several factors may affect adherence to this practice, such as short time to perform tasks, forgetfulness, distance from the sink, lack of observation of attitudes for safe care, dry skin, lack of human resources, lack of knowledge about the need of hand hygiene, inadequate distribution of dispensers and allergy to the products available^19^. In a study carried out in an Intensive Care Unit in the south of Brazil, the lack of materials to perform hand hygiene was considered by the health professionals as the factor with the greatest impact on non-adherence to this measure^25^. 

The structural evaluation carried out for the present study evidenced that there was no shortage of material to perform the actions observed. However, the practice of hand hygiene still had low adherence among healthcare professionals. This scenario is concerning because this is the simplest and most effective measure to ensure patient safety.

It should be noted that, among the specific actions, the use of gloves and the disposal of needles and syringes in the sharps bin had higher adherence than other types of practices. As in other studies^25^ ^-^ ^26^, there was, on the one hand, lower adherence to practices that offer protection to the patient and, on the other hand, a greater concern of the professionals with their own safety, so they gave more attention to personal protective measures. 

This fact may be caused by fear of contamination or occupational diseases. The administration of drugs and the inappropriate disposal of sharp materials can lead to more occurrences of accidents and exposure to biological materials. In this regard, this fear is the main reason for the use of protective equipment, indicating a permanent attitude of care towards oneself^27^. 

However, despite the fact that these actions have high adherence, all practices that involve the safety of the professional must be associated with the care related to patient protection. Thus, to ensure a safe care and reduce risks, all the steps of a care process, and not just some, must be carried out.

It was possible to perceive that a great number of actions related to the Nursing work process were classified as borderline or undesirable. This may be related to high workload, lack of professional experience, inadequate supervision, errors not reported, distractions, non-effective communication, haste and fatigue. In addition, some factors that affect the teams can be highlighted, such as the social and occupational profile, overcrowding in the units and inadequate architecture of the infrastructure^28^.

Regarding the association between work shift and procedure observed, a study with the purpose of identifying errors in the preparation and administration of medications by the nursing team concluded that the greatest number of nonconformities occurred during day shifts^29^. In the present study, the specific actions had similar adherence in the three work shifts, so there was little association between these variables.

The results presented are directly related to institutional specificities, a fact that limits generalization. Among the possible factors that can be related to the existence of shortcomings in the care process assessed, we can cite the high number of admissions, hospitalized patients, procedures and medications and the short staffing of nursing professionals. Despite the data observed, these factors do not justify the results found, since ethics and good professional practices must always be present in the care provided by the nursing team, ensuring a safe care for the patient.

## Conclusion

The evaluation of the nursing practices involving drug administration led to the identification of potentialities and vulnerabilities in the process evaluated. Regarding general conformity, in none of the procedures observed did the professional perform all actions necessary to ensure patient safety, so the practices were classified as undesirable or poor. 

The actions that presented the best positivity index were the use of procedure gloves and sterile materials, in addition to the proper disposal of sharp materials. Among the actions with negative indexes, hand hygiene and disinfection of ampoules and vials were considered as undesirable or poor practices. On the other hand, a greater concern of the professionals with their own security was observed. 

The association between the variables (professional category, work shift and gender) and the procedures observed showed no significant differences. Even though some isolated actions were classified as safe, failure or absence of one of the steps of the procedure compromised the whole care practice. 

Given the above, the results can be considered extremely relevant. From this perspective, the qualification of the team considering local and regional specificities and needs is an important factor for preventing errors and adverse events. Hand hygiene and disinfection of ampoules and injectors with alcohol gel are simple measures to control infections associated with the use of the central vascular catheter, and should be inserted in the care practices.

By implementing these measures, it is possible to develop and implement a safety culture in multi-professional activities, especially nursing care activities. 

In addition, these results are expected to contribute to the development of studies that can produce scientific evidence to enable better nursing care practices and construct a safety culture, favoring policies and programs in the area of patient safety.
